# The role of long-term hair steroids as diagnostic and intervention-related biomarkers in a multimorbid inpatient sample with posttraumatic stress disorder

**DOI:** 10.1080/20008066.2025.2457295

**Published:** 2025-02-24

**Authors:** Lorika Shkreli, Marcella L. Woud, Luisa Bergunde, Lena Schindler-Gmelch, Simon E. Blackwell, Clemens Kirschbaum, Henrik Kessler, Susann Steudte-Schmiedgen

**Affiliations:** a Department of Psychiatry, University of Oxford, UK; b Department of Clinical Psychology and Experimental Psychopathology, Georg-Elias-Mueller-Institute of Psychology, University of Göttingen, Göttingen, Germany; c Mental Health Research and Treatment Center, Ruhr-Universität Bochum, Germany; d Department of Psychotherapy and Psychosomatic Medicine, Faculty of Medicine and University Hospital Carl Gustav Carus, TUD Dresden University of Technology, Dresden, Germany; e Institute and Policlinic of Occupational and Social Medicine, Faculty of Medicine and University Hospital Carl Gustav Carus, TUD Dresden University of Technology, Dresden, Germany; f Department of Clinical Psychology and Psychotherapy, Friedrich-Alexander-Universität Erlangen-Nürnberg, Germany; g Institute of Biological Psychology, Faculty of Psychology, Technische Universität Dresden, Dresden, Germany; h Department of Psychosomatic Medicine and Psychotherapy, LWL-University Hospital, Ruhr Universität Bochum, Bochum, Germany; i Department of Psychosomatic Medicine and Psychotherapy, Fulda Hospital, University Medicine Marburg Campus Fulda, Fulda, Germany

**Keywords:** Treatment response, trauma, biomarker, cortisol, cortisone, DHEA, dehydroepiandrosterone, Respuesta al tratamiento, trauma, biomarcador, cortisol, DHEA, dehidroepiandrosterona

## Abstract

**Background:** Steroid hormone dysregulations have frequently been implicated in posttraumatic stress disorder (PTSD) pathogenesis. However, the translation into naturalistic clinical settings as markers of symptomatology and treatment success remains complex. Particularly, there is little longitudinal data on steroid secretion over the course of interventions.

**Objective:** This study examined the potential of long-term steroid hormone secretion assessed in hair as diagnostic and intervention-related biomarkers among medicated, multimorbid inpatients with PTSD.

**Method:** As part of a secondary analysis of a randomised controlled trial, 54 female inpatients with a primary diagnosis of PTSD received standardised treatment and provided hair samples at pre-treatment, post-treatment, and 3-month follow-up. Cortisol, cortisone, and dehydroepiandrosterone (DHEA) were determined, alongside clinical assessments.

**Results:** Cross-sectional results showed a negative association of pre-treatment DHEA with anxiety symptoms and a trend-level association with lifetime trauma exposure. While inpatients improved in PTSD symptomatology during treatment, neither pre-treatment steroids, nor treatment-induced steroid changes predicted PTSD symptoms at post-treatment or 3-month follow-up.

**Conclusion:** The study highlights the challenges of establishing biomarkers in naturalistic clinical populations. While the association of attenuated DHEA with anxiety symptoms warrants further exploration, our data points towards the potential necessity of patient sub-sample selection to understand, and in the long run clinically target, the endocrine mechanisms in PTSD.

## HIGHLIGHTS

- Hair steroids (Cortisol, Cortisone, DHEA) were assessed in inpatients with PTSD.
- Lower pre-treatment DHEA levels were associated with greater anxiety symptoms.
- Hair steroids did not predict treatment response at hospital discharge or 3-month follow-up.

## Abstract (es)

**Antecedentes:** Las desregulaciones en las hormonas esteroideas han sido frecuentemente asociadas a la patogénesis del trastorno de estrés postraumático (TEPT). Sin embargo, resulta complejo plasmarlo en entornos clínicos naturales como marcadores de sintomatología y éxito en el tratamiento. En particular, la información sobre la secreción de esteroides durante el curso de una intervención clínica es escasa.

**Objetivo:** El estudio examino el potencial de evaluar la secreción capilar de hormonas esteroideas a largo plazo como un biomarcador diagnóstico y asociado a la intervención clínica en pacientes hospitalizados con múltiples comorbilidades y TEPT, y medicados.

**Métodos:** Como parte de un análisis de datos secundarios de un ensayo clínico aleatorizado, se tomaron muestras de cabello previos y al terminar el tratamiento, y 3 meses luego de terminar el tratamiento a 54 mujeres hospitalizadas con un diagnóstico principal de TEPT que recibían un tratamiento estandarizado. Además, junto a evaluaciones clínicas, se evaluaron los niveles de cortisol, cortisona y dehidroepiandrosterona (DHEA).

**Resultados:** Los resultados transversales mostraron una asociación negativa entre los niveles previos al tratamiento de la DHEA y los síntomas de ansiedad, además de una asociación a nivel de tendencia con la exposición a trauma a lo largo de la vida. Si bien las pacientes hospitalizadas mejoraban sus síntomas de TEPT durante el tratamiento, ni los niveles de esteroides previos al tratamiento ni sus cambios inducidos por el tratamiento predijeron los síntomas del TEPT al finalizar el tratamiento o al seguimiento.

**Conclusión:** El estudio enfatiza los desafíos para establecer biomarcadores en poblaciones clínicas naturales. Mientras que la asociación de la atenuación de los niveles de DHEA con los síntomas de ansiedad justifican una mayor exploración, nuestra información señala la potencial necesidad de seleccionar submuestras de pacientes para comprender y, a largo plazo, enfocar de forma clínica los mecanismos endocrinos en el TEPT.

## 1. Introduction

Posttraumatic Stress Disorder (PTSD) is a persistent psychiatric condition that can develop after exposure to an extremely distressing, potentially life-threatening event (American Psychiatric Association, 2013). While cognitive-behavioural therapy (CBT) and eye movement desensitisation and reprocessing (EMDR) are considered the most effective treatments for PTSD (Bisson et al., 2013; Lewis et al., 2020), only around half of patients achieve long-term remission, with even lower rates in comorbid depression (Springer et al., 2018). One important approach to improve clinical care is the integration of biological markers to characterise PTSD symptomology, and to predict long-term treatment response – with the ultimate goal of facilitating the development of novel treatment approaches, monitoring current approaches, and identifying factors that help explain individual differences in treatment outcomes.

In this context, previous research has focused on dysregulations in the bodily stress systems, primarily the hypothalamic-pituitary-adrenal (HPA) axis including its end product cortisol, which has been described to be involved in PTSD pathophysiology and treatment response (Daskalakis et al., 2013; Mehta & Binder, 2012; Rasmusson et al., 2017). However, results of biomarker research investigating cortisol concentrations in PTSD have been inconsistent, not only for characterising PTSD cross-sectionally (Meewisse et al., 2007; Speer et al., 2019), but also for predicting treatment response. For example, while some studies suggested a cortisol increase after successful treatment (Olff et al., 2007; Rauch et al., 2020), meta-analytic evidence revealed no effects between cortisol concentrations and treatment response (Schumacher et al., 2018). This heterogeneity in results might be due to a lack of consistent follow-up assessments, albeit recommended (Cuzick, 2023; Schumacher et al., 2018). That is, cortisol-based differences in treatment response might only become evident in the months following clinic discharge, implying an effect on long-term rather than immediate treatment response. Furthermore, this heterogeneity might be attributable to methodological limitations, as most studies use traditional steroid measurements in saliva, urine, or blood, which only capture short-term secretion with time spans of minutes to hours. Moreover, cortisol secretion is highly volatile to acute influences such as circadian rhythm (Smyth et al., 1997), caffeine intake (Lovallo et al., 2005), or exercise (Hill et al., 2008). Since such influences are not systematically accounted for across studies (Schumacher et al., 2018), it not only hampers comparisons between studies, but also makes it difficult to establish reliable diagnostic and intervention-related biomarkers.

The assessment of steroids in hair has become an increasingly popular tool to retrospectively examine long-term steroid secretion as it is assumed to be less affected by acute influences (Gao et al., 2013; Stalder & Kirschbaum, 2012). Given the advantages of this assessment method, accumulating studies have been conducted in the context of PTSD (for reviews, see Schindler-Gmelch et al., 2023; Steudte-Schmiedgen et al., 2016). In general, cross-sectional and prospective findings suggest that in the short-term aftermath of a traumatic event, cortisol secretion is elevated (Schindler-Gmelch et al., 2023). With more temporal distance to the traumatic event, this initial increase may reverse over time below baseline (Steudte-Schmiedgen et al., 2016), although this pattern was not unequivocally confirmed by a recent systematic review (Schindler-Gmelch et al., 2023). Notably, some studies linked hair cortisol concentrations (HCC) to trauma-exposure independent of PTSD diagnosis (Khoury et al., 2019; Schumacher et al., 2022; Steudte et al., 2013), while others found an association with PTSD symptom severity (Sopp et al., 2021; van den Heuvel et al., 2020). From a mechanistic perspective, attenuated secretion of glucocorticoids (i.e. cortisol and cortisone) has been implicated in impaired memory consolidation (de Quervain et al., 2017), while intact memory consolidation is critical for successful exposure-based therapies such as CBT and likely EMDR (Landin-Romero et al., 2018). Together, these findings provide a theoretical framework of how basic endocrine aberrations might underlie individual differences in treatment response, which warrants their further investigation.

To date, there is only one study investigating the predictive effect of HCC in PTSD treatment (Hummel et al., 2021). It was shown that higher pre-treatment HCC predicted greater improvement in global psychological distress from admission to discharge. However, this effect was neither observed from admission to five months post-discharge nor evident for PTSD symptomology. Further, no predictive effect of HCC change over the course of treatment for clinical outcomes was detected. However, it is important to note that this study did not specify whether patients had received exposure-based treatment, potentially explaining the lack of PTSD-specific results.

Beyond cortisol, there is a growing body of evidence that other HPA axis steroids such as cortisone and dehydroepiandrosterone (DHEA) might also be relevant in PTSD pathophysiology (Kothgassner et al., 2021; van Zuiden et al., 2017). While cortisone is an inactive metabolite of cortisol, there are only a few studies using it as a marker in stress-related research to date (Bae et al., 2019; Vanaelst et al., 2013). However, there are indications that cortisone might be a more robust marker of HPA axis activation than cortisol itself (Perogamvros et al., 2010; Stalder et al., 2013). DHEA is a precursor of androgen and estrogen sex hormones and modulates the activity of 11β-hydroxysteroid dehydrogenase (11β-HSD) enzymes, which are responsible for the interconversion between cortisol and cortisone (Balazs et al., 2008). It is suggested that DHEA has opposing physiological effects to cortisol. That is, while long-term cortisol secretion may have neurotoxic effects, DHEA is considered to exert anti-glucocorticoid and neuroprotective effects in human and animal studies (Kamin & Kertes, 2017; Maninger et al., 2009). With regards to PTSD, higher DHEA concentrations were associated with trauma and prolonged stress exposure using traditional measurements (van Zuiden et al., 2017) as well as hair samples (Buchmüller et al., 2020; Schindler et al., 2019), potentially reflecting compensation mechanisms, i.e. higher stress exposure requiring greater neuroprotective mechanisms (Rasmusson et al., 2010).

As the integration of hair cortisone and DHEA in PTSD research is relatively novel, no study has, to our knowledge, investigated their potential associations with treatment response. While we found no study that included traditional cortisone measurements (saliva, blood, urine) to predict treatment response, initial results regarding traditional DHEA measurements are heterogenous. On the one hand, it was shown that DHEA increased in response to PTSD treatment, without a predictive effect of pre-treatment DHEA concentrations (Olff et al., 2007). On the other hand, pre-treatment DHEA to cortisol ratio predicted post-treatment PTSD and anxiety symptoms (Yehuda et al., 2014).

In the context of a real-world clinical setting, we conducted a randomised controlled trial (RCT) involving an ecologically valid multimorbid inpatient sample with a primary diagnosis of PTSD, who received standardised 8-week treatment as part of their inpatient stay, alongside pharmacological treatment. We investigated the efficacy of an add-on intervention targeting dysfunctional PTSD appraisals (i.e. cognitive bias modification training) vs a sham control training, and included HCC assessments over the course of the intervention (Woud et al., 2018; Woud et al., 2021). While patients receiving the appraisal training showed a greater reduction in the primary outcome measures (i.e. post-traumatic cognitions, dysfunctional appraisals, and PTSD symptoms post-treatment), no such effects were observed for HCC (Woud et al., 2021). However, an exploratory analysis across all patients showed a correlation between reductions in HCC and improvements in dysfunctional PTSD appraisals from pre- to post-treatment (Woud et al., 2021).

In the present secondary analysis, we build upon our previous RCT findings and aim to examine the potential of HCC, hair cortisone, and hair DHEA as clinical biomarkers in PTSD. Specifically, the two main objectives are: (i) to examine these steroids as cross-sectional biomarkers for PTSD symptomatology and related clinical outcomes, and (ii) to assess their role as intervention-related biomarkers for PTSD treatment.

Given the reported associations of attenuated HCC with distal trauma exposure (Steudte-Schmiedgen et al., 2016) and elevated DHEA concentrations with higher trauma load (Buchmüller et al., 2020; Schindler et al., 2019), we expected lower HCC, lower cortisone, and higher DHEA to correlate with pre-treatment symptom severity in our cross-sectional analysis. Further, based on the mechanistic perspective of improved memory consolidation via increased glucocorticoid action (de Quervain et al., 2017) and DHEA’s proposed opposing effects to cortisol (Kamin & Kertes, 2017), we hypothesised better treatment response to be predicted by higher pre-treatment HCC and cortisone (and/or greater HCC and cortisone increase) and lower pre-treatment DHEA (and/or greater DHEA decrease). Finally, we included a sub-sample analysis with only those patients receiving exposure-therapy sessions and expected our hypothesised treatment effects to be more pronounced in this group.

## 2. Methods

### 2.1. Participants

Patient data was a sub-sample (*n* = 54) of a larger randomised controlled trial (RCT, *n* = 80; Woud et al., 2021) that took place at the inpatient ward of the Department of Psychosomatic Medicine and Psychotherapy, LWL-University Clinic of Ruhr-Universität Bochum, Germany. It has been approved by the Ethics Committee of the Faculty of Psychology, Ruhr-Universität Bochum (approval no 204) and by the Ethics Committee of the Faculty of Medicine, Ruhr-Universität Bochum (approval no 15-5477). All patients gave written informed consent.

Out of the original *n* = 80 sample from the RCT (for sample size calculations, see Woud et al., 2018), *n* = 54 (67.5%) eligible patients were included in the analyses for this study if they provided hair samples on at least one assessment time point, and were not taking any steroid or hormonal medications apart from oral contraceptives (Feller et al., 2014; Staufenbiel et al., 2015). Since only two men remained eligible, we decided to exclude them due to the relevance of sex for steroid secretion (Stalder et al., 2017). Finally, the sample consisted of *n* = 54 female inpatients with a primary diagnosis of PTSD, and heterogeneous medical and psychiatric comorbidities (but e.g. no acute suicidality or history of psychosis as based on the RCT’s general inclusion/exclusion criteria, see Woud et al., 2018; Woud et al., 2021). For an overview of participant characteristics see Table 1.

**Table 1.** Overview of patients’ baseline demographic and clinical characteristics.

Baseline Characteristics	*M(SD)*	Range
*Demographics*		
Age	40.11 (12.29)	19–58
BMI	31.16 (9.51)	18.93–63.75
Smoking, *n (%)*	26 (48.15)	
≥ 15 cigarettes/day, *n (%)*	15 (27.78)	
*Clinical Measures*		
BAI – Anxiety	31.24 (10.91)	4–55
BDI-II – Depression	35.74 (8.45)	16–52
Trauma-related information	*n* (%)	Range
LEC-5 – Life-time trauma exposure, *M (SD)*	18.57 (7.23)	6–35
PCL-5 – PTSD Symptoms, *M (SD)*	55.72 (8.88)	35–69
Sexual Violence	32 (59.26)	
Physical Violence	11 (20.37)	
Severe Accident	4 (7.41)	
Other	7 (12.96)	
CAPS-5 total – Trauma Severity, *M (SD)*	43.26 (8.77)	26–71
Years since Index Trauma Exposure, *M (SD)*	22.32 (16.31)	.25–51.00
Comorbidities	*n* (%)	Range
*Psychiatric Comorbidities*		
Patients with comorbidities total	54 (100)	
Major Depression	52 (96.30)	
Eating Disorder	8 (14.81)	
Chronic Pain Disorder	7 (12.96)	
Borderline Personality Disorder	6 (11.11)	
Anxiety Disorder	4 (7.41)	
Substance Dependence/Abuse	3 (5.56)	
Attention Deficit Hyperactivity Disorder	2 (3.71)	
Other	5 (9.26)	
Comorbidities per patient, *M (SD)*	2.61 (0.76)	1–4
*Medical Comorbidities*		
Patients with comorbidities total	20 (37.04)	
Hypothyroidism	11 (20.37)	
Hypertension	6 (11.11)	
Asthma	3 (5.56)	
Other	8 (14.81)	
Comorbidities per patient, *M (SD)*	.52 (.77)	0–3
Medication and Treatment	*n (%)*	Range
Patients on any medication total	52 (96.30)	
Medication changes during treatment	36 (66.67)	
*Psychotropic Medication*		
Patients on psychotropic medication	47 (87.04)	
Antidepressants	40 (74.07)	
Quetiapine	11 (20.37)	
Promethazine	6 (11.11)	
Pipamperone	3 (5.56)	
Valproic Acid	3 (5.56)	
Opioids	3 (5.56)	
Lamotrigine	3 (5.56)	
Pregabalin	3 (5.56)	
Other	16 (29.63)	
Medications per patient, *M (SD)*	1.94 (1.25)	0–5
*Non-Psychotropic Medication*		
Patients on non-psychotropic medication	32 (59.26)	
Thyroid medication	16 (29.63)	
Other	26 (48.15)	
Medications per patient, *M (SD)*	1.44 (2.01)	0–11
Having received Exposure Therapy	37 (68.5)	

Patient data is based on a sub-sample analysis (*n* = 54) from an already published randomised controlled trial. Antidepressants include: Selective serotonin reuptake inhibitors (SSRIs), tricyclic antidepressants, monoamine oxidase inhibitor (MAO) inhibitors and atypical antidepressants. Note: BAI: Becks-Anxiety-Inventory; BDI-II: Becks-Depression-Inventory II; LEC-5: Life-events checklist for DSM-5; PCL-5: PTSD Checklist for DSM-5; CAPS-5: Clinician-Administered PTSD Scale for DSM-5

### 2.2. Procedure

In the first week of clinic admission, the German version (Cwik et al., 2020) of the structured clinical interview CAPS-5 (Clinician-Administered PTSD Scale; et al., 2013a) was administered to confirm PTSD diagnosis before participants were enrolled in the RCT. We included data from the three-time points in which hair samples were provided: pre-treatment, post-treatment, and follow-up (3-month post-discharge). While patients completed a variety of measures as part of the RCT (Woud et al., 2018; Woud et al., 2021), we focused on the following in this study: During pre-treatment, we included demographic information, general anxiety and depression symptoms, PTSD symptoms, and lifetime trauma-exposure. At post-treatment and follow-up, only PTSD symptoms were assessed (for an overview of the therapy schedule, see Woud et al., 2018). Patients stayed in the clinic on average for 47.65 days (*SD* = 9.42) and received standardised treatment (for an overview of the therapy schedule, see Woud et al., 2021).

### 2.3. Clinical assessment

#### 2.3.1. Patient characteristics

Demographic information (e.g. sex, age, BMI and smoking) was assessed through self-report using a self-developed questionnaire. Medications, psychiatric and medical comorbidities were based on medical record data of the inpatient stay.

#### 2.3.2. Lifetime trauma exposure

The Life Events Checklist for DSM-5 (LEC-5; et al., 2013b) was administered to measure lifetime trauma exposure. Based on the DSM-5 A Criterion for PTSD diagnosis, participants indicated their level of exposure to 16 potentially traumatic events from ‘happened to me’, ‘witnessed it’, ‘learned about it’, ‘part of my job’, ‘not sure’, and ‘doesn’t apply’. A weighted cumulative score was calculated (happened to me  = 3, witnessed it  = 2, learned about it  = 1; Weis et al., 2022).

#### 2.3.3. PTSD symptoms

PTSD symptoms were assessed using the PTSD checklist for DMS-5 (PCL-5; et al., 2013), which consists of 20 items covering the 20 PTSD symptoms according to the DSM-5 (e.g. ‘Repeated, disturbing, and unwanted memories of the stressful experience’). Participants rated how much they had experienced each symptom during the previous week on a 5-point Likert-scale from 0 = ‘not at all’ to 4 = ‘extremely’.

#### 2.3.4. General anxiety and depression

General anxiety and depression were reflected by the Beck Depression Inventory-II (BDI; Beck et al., 1996) and Beck Anxiety Inventory (BAI; Beck et al., 1988), respectively. Both questionnaires consist of 21 items assessing symptom severity using 4-point Likert scales.

### 2.4. Hair steroid assessment

Steroids (i.e. cortisol, cortisone, DHEA) were measured using a 3mm-diameter hair sample taken from the posterior vertex region of the scalp and stored in aluminium foil, prior to being sent to the biochemical laboratory (Prof. Clemens Kirschbaum) at Technische Universität Dresden, Germany for steroid analysis. From the scalp, a total length of 3 cm was used for steroid hormone quantification, as this reflects 3 months of hormone secretion with an assumed hair growth of 1 cm per month (Wennig, 2000). Samples were analysed following the established protocol for liquid chromatography tandem mass spectrometry (LC-MS/MS; Gao et al., 2013).

### 2.5. Statistical analyses

Data preparation and analysis were conducted using R Statistical Software (v4.2.1; R Core Team, 2022). All statistical tests were based on alpha level = 0.05 on two-sided tests.

For steroid measures below the detection limit (total: cortisol: 6.3%, 9 values; cortisone: 0%, DHEA: 27.1%, 39 values), we used tailored imputation methods based on the amount of non-detectable values for each hormone and best practices in the field. For Cortisol, we used a fixed-value imputation as recommended for non-detectables <15% (US Environmental Protection Agency, 2015) based on the lowest measure at each time point (pre-treatment = 0.30 pg/mg, post-treatment = 0.03 pg/mg, follow-up = 0.24 pg/mg). For DHEA, we used a distribution-based imputation approach which is the gold-standard for dealing with (particularly larger amounts of) non-detectable values (Herbers et al., 2021). For each time point, a lognonimal distribution was estimated using the lnormimp function within the R package lnormimp (Herbers et al., 2021), from which imputation values for the non-detectables were drawn (pre-treatment 30.2%, 16 values; post-treatment 24.5%, 13 values; follow-up 26.3%, 10 values). Within the lnormimp function, the laboratory detection limit for DHEA (1.2 pg/mg) was specified as the lowest measure in our sample. For outliers (*M* ± 3*SD* per biomarker and assessment point), we conducted a fixed-value imputation based on the highest non-outlier measure of each assessment point (cortisol: 2.1%, 3 values; cortisone: 2.8%, 4 values; DHEA: 2.1%, 3 values).

Prior to statistical analysis, all steroid hormone data was log-transformed to address skewness and improve normality. Steroid change over the course of treatment was determined by calculating the difference score first to retain interpretability (post-treatment minus pre-treatment), then adding a constant to ensure the difference score is > 0 (i.e. minimum difference score per steroid hormone + 1), before applying log-transformation.

While the original RCT used a between-subject design, we pooled patients from both RCT groups and treated them as a single group in all statistical analyses. To check the validity of this approach, we initially carried out analyses including an interaction between the steroid hormone level and group. The lack of any significant interactions supported the pooling of the groups and just included ‘training group’ as a main effect to control for between-group differences in post-training outcome measures.

For all cross-sectional associations between steroids and clinical/trauma-specific outcomes, we used Pearson correlations. Since DHEA values were imputed from a lognominal distribution, slight variations emerge each time an imputed value is estimated (Herbers et al., 2021). Thus, to confirm the robustness and reproducibility of our DHEA results, we repeated correlational analyses for significant and trend-level correlations (i.e. *p*-values below .10) by applying inference testing to the mean correlation coefficients based on 5000 imputations.

To investigate changes in steroids and PTSD symptoms over the course of study participation, we fitted a linear mixed model with random intercept for participants and the fixed-factor of interest ‘time’ (3 time points: pre-treatment, post-treatment, follow-up) for PCL-5 score and each steroid hormone. To account for individual clinical differences, ‘treatment duration’ (i.e. total days spent in the inpatient unit) and ‘lifetime trauma-exposure’ reflected by weighted LEC-5 score were added to all models. To control for confounders from the RCT, ‘training group’ was included in all models (see Woud et al., 2021). Lastly, to control for demographic factors previously shown to influence steroid hormone secretion (Stalder et al., 2017), we included ‘BMI’ and ‘age’ into the respective models for each steroid hormone in which their pre-treatment level showed a significant correlation with these variables.

To investigate whether (i) pre-treatment steroid concentrations and (ii) steroid change over the course of treatment predicted PTSD symptoms at post-treatment and follow-up reflected by PCL-5 scores, we fitted two separate linear mixed models per steroid with a random intercept for participants. In both models, the fixed factors of interest were the respective steroid and the steroid x time interaction. The following control predictors were included as described above: ‘time’ (post-treatment, follow-up), ‘treatment duration’, ‘LEC-5 score’, ‘training group’, ‘BMI’, ‘age’, and ‘pre-treatment PCL-5’. To investigate whether prognostic effects on treatment response were limited to or more pronounced in patients receiving exposure-based therapy, analyses were repeated using only this respective sub-sample (*n* = 37).

All linear-mixed models were fitted using the lmer function within the ‘lme4’ package (Douglas et al., 2015), and the maximum-likelihood estimation. For post-hoc analyses on estimated marginal means the package ‘emmeans’ was used (Lenth, 2023).

## 3. Results

### 3.1. General treatment response and steroid hormone characteristics

Patients improved in their PTSD symptoms over the course of treatment, as reflected by a significant main effect of time on PCL-5 score in the linear mixed model (*β *= −8.46, *t*(100.62) = −6.12, *p* < .001). Post-hoc analyses using estimated marginal means revealed a reduction in PCL-5 scores from pre-treatment to post-treatment (pre-treatment: *M *= 55.7, *SD *= 16.02; post-treatment: *M *= 38.2, *SD *= 16.02, *p* < .001), while PTSD symptoms remained stable between post-treatment and follow-up (*p *= .795; follow-up: *M *= 40.0, *SD *= 18.00).

Steroid concentrations remained stable for all steroids and timepoints (all *ps* > .160) as shown by linear mixed models. While cortisol and cortisone were highly correlated across all time points (all *ps* < .001), there was no significant correlation between any glucocorticoid hormone and DHEA (all *ps* > .281, see Table 2). For demographic variables, there was no significant correlation between pre-treatment cortisol or cortisone with BMI (cortisol: *r *= .23, *p *= .103, cortisone: *r *= .24, *p *= .090) or age (cortisol: *r *= .13, *p *= .360, cortisone: *r *= .14, *p *= .311). However, pre-treatment DHEA was significantly correlated with BMI (*r *= .32, *p *= .019, confirmed via inference testing on the mean correlation of 5000 imputations: *r *= .35, *p *= .011) and age (*r *= -.39, *p *= .004, confirmed via inference testing on the mean correlation of 5000 imputations: *r *= -.39, *p *= .004), indicating younger patients and those with higher BMI had higher pre-treatment DHEA concentrations.

**Table 2.** Steroid hormone descriptives and correlations within each time point.

	Descriptives		Correlations [95% CI]			
* *	*M (SD)*	Range	Cortisol		Cortisone	
*Pre-treatment*						
Cortisol	5.21 (6.68)	.30 –25.51				
Cortisone	14.12 (14.25)	1.43–58.29	.90 ***	[.82, .94]		
DHEA	6.72 (7.68)	.15–36.88	−.03	[−.29, .25]	−.07	[−.33, .20]
*Post-treatment*						
Cortisol	4.71 (5.50)	.03–20.95				
Cortisone	12.60 (10.98)	1.38–42.33	.73 ***	[.57, .84]		
DHEA	6.97 (8.02)	.11–35.20	.02	[−.25, .29]	−.15	[−.40, .12]
*Follow-up*						
Cortisol	4.28 (5.66)	.24–25.90				
Cortisone	10.83 (8.21)	.42–35.65	.71 ***	[.50, .84]		
DHEA	10.92 (21.51)	.15–131.72	−.06	[−.38, .26]	−.07	[−.38, 25]

Note. Steroid concentrations are presented in original units (pg/mg). *** *p*<.001.

### 3.2. Pre-treatment steroid associations with clinical and trauma-specific outcomes

Regarding PTSD symptomology, neither pre-treatment cortisol nor cortisone was correlated with pre-treatment PCL-5 score (both *ps* > .492). While there was a significant inverse correlation between pre-treatment DHEA and pre-treatment PCL-5 (*r *= −.28, *p *= .043), it could not be confirmed by inference testing on the mean correlation of 5000 imputations (*r *= −.21, *p *= .128). Similarly, no association between cortisol and cortisone with lifetime trauma-exposure reflected by LEC-5 scores was revealed, respectively (both *ps* > .400). For DHEA, a trend-level inverse association with LEC-5 score was shown (*r *= −.25, *p *= .076), which was confirmed by inference testing on the mean correlation of 5000 imputations (*r *= −.25, *p *= .070). Regarding general depression and anxiety, pre-treatment steroids were not associated with BDI-II depression scores (all *p*s > .142), while patients reporting higher anxiety symptoms on the BAI showed lower pre-treatment DHEA (*r *= −.32, *p *= .018, confirmed via inference testing on the mean correlation of 5000 imputations, *r *= −.28, *p *= .043), but not cortisol and cortisone levels (both *ps* > .254). To further explore whether the DHEA correlations between general anxiety and lifetime trauma exposure measure distinct constructs, we conducted an additional correlation between BAI and LEC-5, showing that the two measures were not associated (*r *= .14, *p *= .303).

### 3.3. Steroid hormones as markers for treatment response

Linear mixed models revealed that neither pre-treatment steroid concentrations, nor steroid change predicted PTSD symptoms at post-treatment or follow-up (main effects: all *ps* > .308, steroid × time interaction effects: all *ps* > .218; see table 3 for parameters). In the sub-sample analysis including only patients having received exposure-based therapy, results remained unchanged (all *ps* > .146; Table 3). For an overview of all full model parameters including control predictors, see supplementary Tables S1 and S2.

**Table 3.** Overview of steroid hormones as intervention-related biomarkers for PTSD treatment, reflected by PTSD symptoms after treatment and follow-up.

	Cortisol				Cortisone				DHEA			
	*B*	*SE*	*95% CI*	*p*	*B*	*SE*	*95% CI*	*p*	*B*	*SE*	*95% CI*	*p*
*Full Sample – Baseline*												
Steroid	3.32	10.25	[−17.00, 23.83]	.747	13.74	13.38	[−12.85, 40.42]	.308	3.36	8.81	[−14.14, 2.09]	.704
Steroid x Time	−3.25	3.85	[−11.07, 4.42]	.403	−6.23	4.98	[−16.26, 3.74]	.218	−.01	3.16	[−6.36, 6.33]	.997
*Full Sample – Change Score*												
Steroid Change	−7.64	41.25	[−90.34, 74.39]	.854	−8.52	25.70	[−59.97, 42.57]	.742	9.35	22.68	[−35.80, 54.42]	.681
Steroid Change x Time	8.75	18.51	[−28.12, 46.02]	.639	8.41	10.93	[−13.38, 30.43]	.446	−7.29	7.90	[−23.11, 8.61]	.361
*Subsample – Baseline*												
Steroid	9.16	12.18	[−15.11, 33.79]	.456	17.59	15.12	[−12.63, 47.95]	.251	5.61	11.21	[−16.78, 28.08]	.619
Steroid x Time	−5.60	4.58	[−15.04, 3.58]	.231	−8.30	5.56	[−19.63, 2.92]	.146	.03	3.96	[−8.03, 8.02]	.995
*Subsample – Change Score*												
Steroid change	4.06	49.13	[−94.66, 103.03]	.935	−3.27	29.01	[−61.64, 54.91]	.911	18.61	115.78	[−213.26, 250.32]	.873
Steroid change x Time	2.03	22.24	[−42.98, 46.89]	.928	4.96	12.25	[−19.76, 29.77]	.689	−51.11	41.04	[−134.35, 32.18]	.223

Note. Models were fitted using linear mixed models with random intercept for participant. The estimates for each steroid hormone, steroid hormone change and their interaction with time are presented. Time has two levels (post-treatment, follow-up). All models were controlled for pre-treatment PTSD symptoms, treatment duration, lifetime trauma exposure and training group. DHEA models were further corrected for age and BMI due to being correlated with these confounding variables. Results are presented for the full sample (*N* = 54) and the subsample receiving exposure-based therapy (*n* = 37). For an overview of estimates of all model parameters including control predictors, see Supplementary Table S1 and S2.

## 4. Discussion

In the present study, we examined whether the steroid hormones cortisol, cortisone, and DHEA assessed from hair samples were associated with clinical symptomatology and treatment response in an ecologically valid multimorbid inpatient PTSD sample with a primary diagnosis of PTSD. Pre-treatment associations between steroids and clinical outcomes showed that lower DHEA was significantly associated with higher general anxiety symptoms, and on a trend-level with greater lifetime trauma exposure. However, neither pre-treatment steroid concentrations, nor their change in response to inpatient treatment showed a predictive effect on PTSD symptomatology.

In our cross-sectional pre-treatment analyses, contrary to our expectations, cortisol and cortisone did not show any associations with trauma-specific outcomes. However, there was a trend-level negative correlation between DHEA and lifetime trauma exposure. Similarly, while there were no associations between cortisol or cortisone with depression or anxiety symptoms, lower DHEA levels were associated with more pronounced anxiety symptoms. Notably, anxiety symptoms and trauma history were uncorrelated.

Our results contrast initial findings reporting no association between hair DHEA and anxiety symptoms in a female sample of Palestinian adolescents living in the West Bank with and without trauma exposure/PTSD (Schindler et al., 2019), and in non-clinical populations (Kische et al., 2023; Renner et al., 2021; Ullmann et al., 2016; Walther et al., 2019).

Since it has been suggested that DHEA has opposing effects to cortisol in being a marker of neuroprotection rather than of stress (Kamin & Kertes, 2017; Maninger et al., 2009), the negative associations between attenuated DHEA levels and clinical measures in our cross-sectional analysis may appear surprising. An opposing pattern to cortisol (i.e. elevated DHEA concentrations) was expected based on previous research in trauma exposed individuals using hair samples (Buchmüller et al., 2020; Schindler et al., 2019) and traditional steroid measures (van Zuiden et al., 2017). However, as we are only beginning to understand the role of DHEA in stress-related research, there is an ongoing discussion as to whether DHEA could be seen as another stress marker with a similar secretion pattern to cortisol, i.e. chronic stress exposure leading to depleted neuroprotective mechanisms reflected by attenuated rather than elevated DHEA concentrations (Kamin & Kertes, 2017; Kanter et al., 2001; Oe et al., 2012). Accordingly, the aforementioned associations between increased hair DHEA concentrations and trauma exposure were reported in refugees and individuals living in current war zones (Buchmüller et al., 2020; Schindler et al., 2019), so that stress exposure might have been too acute to result in attenuated DHEA secretion yet, especially since not all included individuals were diagnosed with PTSD. Overall, our results indicate that DHEA might be a more sensitive HPA axis marker than cortisol in an inpatient sample with a primary diagnosis of PTSD, although targeted research is needed to confirm this suggestion, establish the direction of the association between DHEA concentrations and trauma exposure, as well as disentangle the specific effects of anxiety and PTSD. Further, there is initial evidence suggesting that HCC and hair DHEA might be distinctively associated with specific PTSD symptoms, rather than general trauma exposure (de Graaff et al., 2023). Although the reported associations were small, future studies may wish to include robust measurements of symptom clusters to contribute to a detailed understanding of subtle nuances in HPA-axis response.

Our prognostic null-results on treatment response are in line with a meta-analytic review of traditional cortisol measurements in blood and saliva (Schumacher et al., 2018). Further, the only currently available study using HCC in a comparable inpatient sample did not show a predictive effect on PTSD symptoms, but an association between higher pre-treatment HCC and improvements in general psychopathology (Hummel et al., 2021). This is comparable to a study by Fischer et al. (2018) demonstrating in a clinical anxiety and depression population that treatment responders (vs non-responders) had higher pre-treatment HCC. However, it is unknown whether all patients in the study by Hummel et al. (2021) received exposure-based treatment. This might explain the lack of PTSD-specific results, considering the assumptions that the key underlying mechanism of exposure-based treatment is memory consolidation which is facilitated by glucocorticoids (de Quervain et al., 2017). However, to better understand this finding, we repeated our analyses in a sub-sample of patients (*n* = 37) receiving exposure-based treatment and the results remained unchanged. This indicates that hair steroids may, at least in our multimorbid sample, not be suited as a biomarker for PTSD treatment response. Notably, due to the smaller size of the subsample, the statistical power to detect relevant effects might have been insufficient, highlighting the importance of targeted research in this sub-group for future investigations.

One of the reasons for the lack of cross-sectional and intervention-related effects on PTSD symptoms might be that a variety of confounding factors were present in our sample, which exert distinct effects on HPA axis functioning. To illustrate, the study patients had several medical and psychiatric comorbidities, while their prescribed medication changed throughout their inpatient care. Most notably, the majority of patients were diagnosed with comorbid major depression and regularly consumed nicotine, both factors that have been repeatedly associated with changes in the HPA axis (Feller et al., 2014; Pochigaeva et al., 2017; Staufenbiel et al., 2013, but see Psarraki et al., 2021). Similarly, preliminary evidence points towards hormonal contraception and thyroid medication affecting HPA axis activity (Feller et al., 2014), which we could also not account for. This complexity is heightened by the fact that psychotropic medications like antidepressant and antipsychotics, while generally reducing steroid levels (Romanova et al., 2022; Subramaniam et al., 2019), may have varying effects depending on the patient’s baseline cortisol levels and treatment response, making it challenging to isolate medication effects in a diverse clinical population. However, while clearly a challenge in terms of robust steroid concentrations, these characteristics are typical of inpatients with PTSD in clinical settings, thereby simultaneously representing one of the greatest strengths of this study – the high ecological validity. While most research in HPA axis activity focuses on excluding confounding factors like comorbid diagnoses or certain medications, it is particularly relevant to design studies allowing for their interplay, especially when aiming to establish a biomarker. As clinical conditions rarely occur isolated in a real-world clinical setting, an ideal biomarker needs to be robust to such confounding variables and pronounced enough to not only be visible in extremely large datasets. Otherwise, its translation into clinical settings is extremely limited. While it is important in the very first step to understand whether there is an association between a biomarker and a psychiatric condition in a highly selective sample, it is equally important to take the next step and observe how this biomarker fares in a naturalistic population. The biggest challenge here is to find the right balance between using an unfiltered clinical population, which limits drawing systematic conclusions in case of null-results and creating non-representative research patient samples which are not encountered in everyday clinical care. It might be useful in future studies to systematically cluster patients with PTSD based on the most common combinations of comorbidities and confounding factors, to then investigate whether hair steroids are sensitive in predicting treatment response within those sub-groups. Moreover, it has been proposed to consider the inclusion of additional HPA axis parameters, such as glucocorticoid receptor status or binding protein levels, as a single biomarker might not be sufficient to predict treatment response (Stalder et al., 2017).

In addition, our study’s relatively small sample size may limit the detection of nuanced steroid effects on PTSD symptomatology or treatment response, particularly in such a complex clinical population. While similar null results on HCC were reported in a comparable PTSD inpatient study of similar sample size (Hummel et al., 2021), a study by Dajani et al. (2018), with over 700 war-exposed participants, used trajectory modelling to show that HCC normalised after a psychosocial intervention. Thus, larger sample sizes, allowing for more refined analytical approaches, may be necessary to capture HCC effects in complex clinical populations. A multicentre RCT investigating the effectiveness of two PTSD interventions, which also incorporate HCC measurements, is currently in progress and will provide more comprehensive insights into the relationship between PTSD treatments and HCC in a large outpatient sample (Leichsenring et al., 2020).

Notably, while the PCL-5 scores decreased significantly from baseline, participants on average remained above the clinical cut-off for PTSD. This pattern is comparable to other studies in inpatient PTSD settings (e.g. Hummel et al., 2021), and suggests that predictive effects of hair steroids may only become evident when patients achieve greater symptom improvement.

This study has some limitations that need to be considered. Firstly, our sample combined patients from the two training groups of the original RCT (Woud et al., 2021). Therefore, even after statistical control, we cannot fully rule out that the trauma-appraisal training might have influenced steroid secretion or biased estimation of treatment outcomes. While our findings indicate that hair steroid hormone levels were not associated with PTSD symptomatology or treatment response, a key limitation is the lack of established reference values for steroid hormones in hair, especially in clinical populations. The impact of such limitation becomes particularly important and noticeable in the interpretation of the high proportion of non-detectable DHEA values (27.1%) despite using the gold-standard assessment method (LC-MS/MS). It remains unclear whether such low values are a result of clinical conditions like PTSD or due to the effects of psychotropic medication, which could have further reduced steroid hormone levels (Romanova et al., 2022; Subramaniam et al., 2019). Thus, more fundamental research on the potential influence of psychotropic medication on steroid hormone levels are factors that warrant further investigation in future studies. Similarly, more research is needed to establish baseline steroid hormone concentrations in clinical populations and refine analytical methods to improve detection accuracy.

In addition, given the lack of a healthy control group, we could not account for time-dependent steroid changes due to external factors, such as seasonal variations, nor assess baseline differences in steroid concentrations between patients and healthy individuals. Looking ahead, it might be useful to further investigate how steroids are associated with cognitive markers of PTSD treatment response, as initiated in our original RCT, to gain mechanistic insights relevant to CBT improvement.

## 5. Conclusion

Overall, this study indicates that the steroids cortisol, cortisone, and DHEA measured in hair do not serve as robust biomarkers for PTSD symptomatology and treatment response, at least in the present medicated and multimorbid inpatient sample. Targeted research is needed to explore whether long-term endocrine biomarkers might be more suitable in specific patient sub-groups, or whether the inclusion of complementing HPA axis parameters (e.g. glucocorticoid receptor status or stress reactivity) might be beneficial.

## Supplementary Material

Shkreli_et_al_Supplementary_Material_EJPT.docx

Shkreli_et_al_revised_Supplementary_Material_EJPT.docx

## Data Availability

Anonymised outcome data were based on the RCT by Woud et al. (2021) and are available at: https://osf.io/jvstf/.
