# Supplementary material for: The role of long-term hair steroids as diagnostic and intervention-related biomarkers in a multimorbid inpatient sample with posttraumatic stress disorder
Source: Eur J Psychotraumatol. 2025 Feb 24;16(1):2457295. doi: 10.1080/20008066.2025.2457295 (PMC11852232; doi:10.1080/20008066.2025.2457295)
Supplement: Shkreli_et_al_Supplementary_Material_EJPT.docx [file ZEPT_A_2457295_SM8479.docx]

**Supplementary Material:** **The role of long-term hair steroids as diagnostic and intervention-related markers in a multimorbid inpatient sample with posttraumatic stress disorder**

**Table S1.** Full sample (N=54): Overview of all estimates within the linear mixed models predicting PTSD symptoms post-treatment and follow up

|  |  |  | Cortisol |  |  |  |  |  | Cortisone |  |  |  |  |  | DHEA |  |  |
| --- | --- | --- | --- | --- | --- | --- | --- | --- | --- | --- | --- | --- | --- | --- | --- | --- | --- |
|  | *B* | *SE* | *95 % CI* | *p* |  |  | *B* | *SE* | *95 % CI* | *p* |  |  | *B* | *SE* | *95 % CI* | *p* |  |
| *Pre-treatment steroid* |  |  |  |  |  |  |  |  |  |  |  |  |  |  |  |  |  |
| BMI | -.45 | .26 | [-.97, .078] | .096 |  |  | -.49 | .26 | [-1.01, .04] | .070 |  |  | -.54 | .27 | [-1.09, -8.50] | .051 |  |
| Age | -.03 | .21 | [-.44, .38] | .889 |  |  | -.05 | .21 | [-.47, .37] | .810 |  |  | .03 | .22 | [-.41, 4.67] | .902 |  |
| Pre-treatment PCL-5 | .07 | .30 | [.11, 1.29] | .021 | * |  | .70 | .30 | [.11, 1.30] | .022 | * |  | .72 | .30 | [.12, 1.31] | .020 | * |
| Treatment duration | -.27 | .27 | [-.81, .27] | .331 |  |  | -.26 | .27 | [-.80, .29] | .351 |  |  | -.27 | .27 | [-.81, 2.70] | .323 |  |
| LEC-5 | .51 | .36 | [-.21, 1.24] | .161 |  |  | .56 | .36 | [-.17, 1.29] | .133 |  |  | .56 | .36 | [-.16, 1.29] | .127 |  |
| Training group | .17 | 5.20 | [-10.20, 10.55] | .974 |  |  | -.84 | 4.99 | [-10.79, 9.14] | .867 |  |  | -.92 | 4.96 | [-10.81, 9.01] | .854 |  |
| Time | 5.32 | 5.73 | [-6.06, 16.97] | .358 |  |  | 12.76 | 9.89 | [-7.00, 32.70] | .204 |  |  | .74 | 5.26 | [-9.76, 1.31] | .889 |  |
| Steroid | 5.10 | 10.23 | [-15.20, 25.58] | .620 |  |  | 16.27 | 13.39 | [-10.35, 42.96] | .229 |  |  | 3.36 | 8.81 | [-14.14, 2.09] | .704 |  |
| Steroid x time | -3.23 | 3.84 | [-11.02, 4.41] | .405 |  |  | -6.14 | 4.97 | [-16.13, 3.80] | .223 |  |  | -.01 | 3.16 | [-6.36, 6.33] | .997 |  |
| *Steroid change* |  |  |  |  |  |  |  |  |  |  |  |  |  |  |  |  |  |
| BMI | -.48 | .26 | [-1.00, .029] | .066 |  |  | -.45 | .26 | [-.97, .06] | .080 |  |  | -.49 | .26 | [-1.01, .03] | .065 |  |
| Age | .01 | .21 | [-.41, .43] | .961 |  |  | -.01 | .21 | [-.41, 44] | .945 |  |  | -.03 | .21 | [-.45, .38] | .880 |  |
| Pre-treatment PCL-5 | .66 | .30 | [.07, 1.26] | .030 | * |  | .68 | .30 | [.09, 1.27] | .025 | * |  | .75 | .31 | [.14, 1.37] | .018 |  |
| Treatment duration | -.26 | .27 | [-.80, .28] | .347 |  |  | -.27 | .27 | [-.81, .27] | .324 |  |  | -.30 | .27 | [-.84, .25] | .282 |  |
| LEC-5 | .50 | .36 | [-.22, 1.22] | .172 |  |  | .49 | .36 | [-.23, 1.22] | .179 |  |  | .53 | .36 | [-.18, 1.25] | .141 |  |
| Training group | -.61 | 4.94 | [-10.44, 9.26] | .902 |  |  | -.67 | 4.93 | [-10.51, 9.19] | .892 |  |  | .02 | 5.03 | [-10.00, 10.06] | .997 |  |
| Time | -14.49 | 40.86 | [-96.33, 67.16] | .725 |  |  | -16.01 | 26.28 | [-68.59, 36.63] | .545 |  |  | 18.21 | 19.02 | [-20.04, 56.33] | .344 |  |
| Steroid change | -2.66 | 41.14 | [-84.83, 79.33] | .949 |  |  | -6.15 | 25.69 | [-57.36, 45.07] | .812 |  |  | 9.35 | 22.68 | [-35.80, 54.42] | .681 |  |
| Steroid change x time | 6.87 | 18.44 | [-29.97, 43.82] | .711 |  |  | 6.96 | 10.91 | [-14.89, 28.82] | .530 |  |  | -7.29 | 7.90 | [-23.11, 8.61] | .361 |  |

Time has two levels (post-treatment, follow-up).

Note: PCL-5: PTSD Checklist for DSM-5; LEC-5: Life-events checklist for DSM-5

**Table S2.** Exposure-based subsample (n=37): Overview of all estimates within the linear mixed models predicting PTSD symptoms post-treatment and follow up. ‘Time’ has two levels (post-treatment and follow-up).

|  |  |  | Cortisol |  |  |  |  |  | Cortisone |  |  |  |  |  | DHEA |  |  |
| --- | --- | --- | --- | --- | --- | --- | --- | --- | --- | --- | --- | --- | --- | --- | --- | --- | --- |
|  | *B* | *SE* | *95 % CI* | *p* |  |  | *B* | *SE* | *95 % CI* | *p* |  |  | *B* | *SE* | *95 % CI* | *p* |  |
| *Pre-treatment steroid* |  |  |  |  |  |  |  |  |  |  |  |  |  |  |  |  |  |
| BMI | -.37 | .40 | [-1.17, .43] | .358 |  |  | -.43 | .40 | [-1.24, .38] | .290 |  |  | -.53 | .40 | [-1.33, .27] | .193 |  |
| Age | -.20 | .30 | [-.82, .41] | .509 |  |  | -.22 | .31 | [-.84, .39] | .472 |  |  | -.07 | .33 | [-.73, .59] | .842 |  |
| Pre-treatment PCL-5 | .62 | .43 | [-.25, 1.48] | .158 |  |  | .61 | .43 | [-.26, 1.47] | .165 |  |  | .58 | .43 | [-.27, 1.44] | .179 |  |
| Treatment duration | -.16 | .36 | [-.88, .57] | .667 |  |  | -.17 | .36 | [-.90, .55] | .637 |  |  | -.15 | .36 | [-.87, .58] | .685 |  |
| LEC-5 | .67 | .52 | [-.36, 1.71] | .199 |  |  | .76 | .52 | [-.28, 1.81] | .150 |  |  | -.70 | .48 | [-.27, 1.68] | .156 |  |
| Training group | .91 | 6.77 | [-12.68, 14.57] | .893 |  |  | .13 | 6.48 | [-12.87, 13.20] | .984 |  |  | -.02 | 6.42 | [-12.91, 12.05] | .998 |  |
| Time | 8.76 | 6.97 | [-5.17, 23.18] | .219 |  |  | 17.26 | 11.18 | [-5.22, 40.12] | .133 |  |  | .55 | 6.23 | [-12.00, 13.29] | .930 |  |
| Steroid | 11.69 | 12.26 | [-12.74, 36.46] | .345 |  |  | 21.83 | 15.30 | [-8.72, 52.54] | .160 |  |  | 5.61 | 11.21 | [-16.78, 28.08] | .619 |  |
| Steroid x time | -5.69 | 4.58 | [-15.14, 3.48] | .224 |  |  | -8.47 | 5.56 | [-19.81, 2.73] | .138 |  |  | .03 | 3.96 | [-8.03, 8.02] | .995 |  |
| *Steroid change* |  |  |  |  |  |  |  |  |  |  |  |  |  |  |  |  |  |
| BMI | -.42 | .38 | [-1.19, .35] | .283 |  |  | -.40 | .39 | [-1.17, .38] | .312 |  |  | -.53 | .37 | [-1.29, .22] | .160 |  |
| Age | -.15 | .32 | [-.80, .49] | .634 |  |  | -.16 | .32 | [-.80, .49] | .626 |  |  | -.16 | .29 | [-.75, .43] | .594 |  |
| Pre-treatment PCL-5 | .55 | .44 | [-.34, 1.45] | .220 |  |  | .56 | .44 | [-.33, 1.45] | .214 |  |  | .55 | .41 | [-.28, 1.38] | .193 |  |
| Treatment duration | -.18 | .36 | [-.91, .55] | .623 |  |  | -.19 | .36 | [-.92, .54] | .607 |  |  | -.31 | .35 | [-1.02, .40] | .385 |  |
| LEC-5 | .67 | .50 | [-.33, 1.67] | .186 |  |  | .68 | .50 | [-.32, 1.67] | .182 |  |  | .58 | .47 | [-.37, 1.53] | .227 |  |
| Training group | .47 | 6.48 | [-12.53, 13.55] | .943 |  |  | .47 | 6.48 | [-12.53, 13.55] | .943 |  |  | -2.80 | 6.51 | [-15.86, 10.34] | .670 |  |
| Time | -1.03 | 48.80 | [-99.08, 98.12] | .983 |  |  | -9.51 | 29.17 | [-68.38, 49.62] | .747 |  |  | 125.02 | 99.90 | [-77.64, 327.74] | .221 |  |
| Steroid change | 3.91 | 49.19 | [-94.68, 103.25] | .937 |  |  | -5.16 | 29.20 | [-63.74, 53.50] | .860 |  |  | 18.61 | 115.78 | [-213.26, 250.32] | .873 |  |
| Steroid change x time | .72 | 22.21 | [-44.38, 45.36] | .974 |  |  | 4.24 | 12.25 | [-20.58, 28.98] | .732 |  |  | -51.11 | 41.04 | [-134.35, 32.18] | .223 |  |

Note: PCL-5: PTSD Checklist for DSM-5; LEC-5: Life-events checklist for DSM-5
