# Supplementary material for: The role of long-term hair steroids as diagnostic and intervention-related biomarkers in a multimorbid inpatient sample with posttraumatic stress disorder
Source: Eur J Psychotraumatol. 2025 Feb 24;16(1):2457295. doi: 10.1080/20008066.2025.2457295 (PMC11852232; doi:10.1080/20008066.2025.2457295)
Supplement: Shkreli_et_al_revised_Supplementary_Material_EJPT.docx [file ZEPT_A_2457295_SM8478.docx]

**Supplementary Material:** **The role of long-term hair steroids as diagnostic and intervention-related markers in a multimorbid inpatient sample with posttraumatic stress disorder**

**Table S1.** Full sample (N=54): Overview of all estimates within the linear mixed models predicting PTSD symptoms post-treatment and follow up

|  |  |  | Cortisol |  |  |  |  |  | Cortisone |  |  |  |  |  | DHEA |  |  |
| --- | --- | --- | --- | --- | --- | --- | --- | --- | --- | --- | --- | --- | --- | --- | --- | --- | --- |
|  | *B* | *SE* | *95 % CI* | *p* |  |  | *B* | *SE* | *95 % CI* | *p* |  |  | *B* | *SE* | *95 % CI* | *p* |  |
| *Pre-treatment steroid* |  |  |  |  |  |  |  |  |  |  |  |  |  |  |  |  |  |
| BMI |  |  |  |  |  |  |  |  |  |  |  |  | -.54 | .27 | [-1.09, -8.50] | .051 |  |
| Age |  |  |  |  |  |  |  |  |  |  |  |  | .03 | .22 | [-.41, 4.67] | .902 |  |
| Pre-treatment PCL-5 | .76 | .30 | [.18, 1.34] | .012 | * |  | .75 | .29 | [.17, 1.34] | .013 | * |  | .72 | .30 | [.12, 1.31] | .020 | * |
| Treatment duration | -.13 | .26 | [-.66, .39] | .621 |  |  | -.12 | .27 | [-.65, .42] | .667 |  |  | -.27 | .27 | [-.81, 2.70] | .323 |  |
| LEC-5 | .45 | .36 | [-.27, 1.17] | .221 |  |  | .47 | .36 | [-.25, 1.20] | .199 |  |  | .56 | .36 | [-.16, 1.29] | .127 |  |
| Training group | .77 | 5.13 | [-9.47, 11.02] | .880 |  |  | -.70 | 4.94 | [-10.56, 9.17] | .888 |  |  | -.92 | 4.96 | [-10.81, 9.01] | .854 |  |
| Time | 5.35 | 5.74 | [-6.06, 17.06] | .357 |  |  | 12.92 | 9.92 | [-6.89, 32.94] | .200 |  |  | .74 | 5.26 | [-9.76, 1.31] | .889 |  |
| Steroid | 3.32 | 10.25 | [-17.00, 23.83] | .747 |  |  | 13.74 | 13.38 | [-12.85, 40.42] | .308 |  |  | 3.36 | 8.81 | [-14.14, 2.09] | .704 |  |
| Steroid x time | -3.25 | 3.85 | [-11.07, 4.42] | .403 |  |  | -6.23 | 4.98 | [-16.26, 3.74] | .218 |  |  | -.01 | 3.16 | [-6.36, 6.33] | .997 |  |
| *Steroid change* |  |  |  |  |  |  |  |  |  |  |  |  |  |  |  |  |  |
| BMI |  |  |  |  |  |  |  |  |  |  |  |  | -.49 | .26 | [-1.01, .03] | .065 |  |
| Age |  |  |  |  |  |  |  |  |  |  |  |  | -.03 | .21 | [-.45, .38] | .880 |  |
| Pre-treatment PCL-5 | .74 | .29 | [.16, 1.32] | .014 | * |  | .76 | .29 | [.18, 1.34] | .020 | * |  | .75 | .31 | [.14, 1.37] | .018 |  |
| Treatment duration | -.10 | .26 | [-.63, .43] | .706 |  |  | -.12 | .26 | [-.64, .40] | .649 |  |  | -.30 | .27 | [-.84, .25] | .282 |  |
| LEC-5 | .46 | .36 | [-.26, 1.18] | .207 |  |  | .45 | .36 | [-.26, 1.17] | .213 |  |  | .53 | .36 | [-.18, 1.25] | .141 |  |
| Training group | -.40 | 4.88 | [-10.13, 9.35] | .936 |  |  | -.39 | 4.86 | [-10.08, 9.32] | .937 |  |  | .02 | 5.03 | [-10.00, 10.06] | .997 |  |
| Time | -18.66 | 41.02 | [-101.20, 63.07] | .651 |  |  | -19.49 | 26.32 | [-72.47, 32.99] | .463 |  |  | 18.21 | 19.02 | [-20.04, 56.33] | .344 |  |
| Steroid change | -7.64 | 41.25 | [-90.34, 74.39] | .854 |  |  | -8.52 | 25.70 | [-59.97, 42.57] | .742 |  |  | 9.35 | 22.68 | [-35.80, 54.42] | .681 |  |
| Steroid change x time | 8.75 | 18.51 | [-28.12, 46.02] | .639 |  |  | 8.41 | 10.93 | [-13.38, 30.43] | .446 |  |  | -7.29 | 7.90 | [-23.11, 8.61] | .361 |  |

Time has two levels (post-treatment, follow-up).

Note: PCL-5: PTSD Checklist for DSM-5; LEC-5: Life-events checklist for DSM-5

**Table S2.** Exposure-based subsample (n=37): Overview of all estimates within the linear mixed models predicting PTSD symptoms post-treatment and follow up. ‘Time’ has two levels (post-treatment and follow-up).

|  |  |  | Cortisol |  |  |  |  |  | Cortisone |  |  |  |  |  | DHEA |  |  |
| --- | --- | --- | --- | --- | --- | --- | --- | --- | --- | --- | --- | --- | --- | --- | --- | --- | --- |
|  | *B* | *SE* | *95 % CI* | *p* |  |  | *B* | *SE* | *95 % CI* | *p* |  |  | *B* | *SE* | *95 % CI* | *p* |  |
| *Pre-treatment steroid* |  |  |  |  |  |  |  |  |  |  |  |  |  |  |  |  |  |
| BMI |  |  |  |  |  |  |  |  |  |  |  |  | -.53 | .40 | [-1.33, .27] | .193 |  |
| Age |  |  |  |  |  |  |  |  |  |  |  |  | -.07 | .33 | [-.73, .59] | .842 |  |
| Pre-treatment PCL-5 | .59 | .43 | [-.27, 1.46] | .176 |  |  | .56 | .43 | [-.32, 1.43] | .207 |  |  | .58 | .43 | [-.27, 1.44] | .179 |  |
| Treatment duration | -.03 | .32 | [-.69, .63] | .927 |  |  | -.03 | .33 | [-.70, .64] | .928 |  |  | -.15 | .36 | [-.87, .58] | .685 |  |
| LEC-5 | .42 | .49 | [-.55, 1.41] | .384 |  |  | .47 | .49 | [-.51, 1.46] | .337 |  |  | -.70 | .48 | [-.27, 1.68] | .156 |  |
| Training group | 1.40 | 6.56 | [-11.77, 14.62] | .832 |  |  | .10 | 6.33 | [-12.62, 12.99] | .987 |  |  | -.02 | 6.42 | [-12.91, 12.05] | .998 |  |
| Time | 8.61 | 6.98 | [-5.34, 23.04] | .227 |  |  | 16.90 | 11.19 | [-5.64, 39.75] | .142 |  |  | .55 | 6.23 | [-12.00, 13.29] | .930 |  |
| Steroid | 9.16 | 12.18 | [-15.11, 33.79] | .456 |  |  | 17.59 | 15.12 | [-12.63, 47.95] | .251 |  |  | 5.61 | 11.21 | [-16.78, 28.08] | .619 |  |
| Steroid x time | -5.60 | 4.58 | [-15.04, 3.58] | .231 |  |  | -8.30 | 5.56 | [-19.63, 2.92] | .146 |  |  | .03 | 3.96 | [-8.03, 8.02] | .995 |  |
| *Steroid change* |  |  |  |  |  |  |  |  |  |  |  |  |  |  |  |  |  |
| BMI |  |  |  |  |  |  |  |  |  |  |  |  | -.53 | .37 | [-1.29, .22] | .160 |  |
| Age |  |  |  |  |  |  |  |  |  |  |  |  | -.16 | .29 | [-.75, .43] | .594 |  |
| Pre-treatment PCL-5 | .51 | .44 | [-.37, 1.39] | .251 |  |  | .51 | .44 | [-.37, 1.39] | .253 |  |  | .55 | .41 | [-.28, 1.38] | .193 |  |
| Treatment duration | -.02 | .33 | [-.69, .65] | .958 |  |  | -.04 | .34 | [-.71, .62] | .890 |  |  | -.31 | .35 | [-1.02, .40] | .385 |  |
| LEC-5 | .46 | .47 | [-.50, 1.42] | .340 |  |  | .47 | .48 | [-.49, 1.43] | .326 |  |  | .58 | .47 | [-.37, 1.53] | .227 |  |
| Training group | .67 | 6.35 | [-12.08, 13.49] | .916 |  |  | .66 | 6.33 | [-12.06, 13.43] | .918 |  |  | -2.80 | 6.51 | [-15.86, 10.34] | .670 |  |
| Time | -3.95 | 48.87 | [-102.46, 94.99] | .936 |  |  | -11.26 | 29.16 | [-70.30, 47.63] | .702 |  |  | 125.02 | 99.90 | [-77.64, 327.74] | .221 |  |
| Steroid change | 4.06 | 49.13 | [-94.66, 103.03] | .935 |  |  | -3.27 | 29.01 | [-61.64, 54.91] | .911 |  |  | 18.61 | 115.78 | [-213.26, 250.32] | .873 |  |
| Steroid change x time | 2.03 | 22.24 | [-42.98, 46.89] | .928 |  |  | 4.96 | 12.25 | [-19.76, 29.77] | .689 |  |  | -51.11 | 41.04 | [-134.35, 32.18] | .223 |  |

Note: PCL-5: PTSD Checklist for DSM-5; LEC-5: Life-events checklist for DSM-5
